# Toxic ignorance and right-to-know in biomonitoring results communication: a survey of scientists and study participants

**DOI:** 10.1186/1476-069X-8-6

**Published:** 2009-02-28

**Authors:** Rachel Morello-Frosch, Julia Green Brody, Phil Brown, Rebecca Gasior Altman, Ruthann A Rudel, Carla Pérez

**Affiliations:** 1 Department of Environmental Science, Policy and Management & School of Public Health, 137 Mulford Hall, University of California, Berkeley, Berkeley, CA 94720-3114, USA; 2 Silent Spring Institute, 29 Crafts Street, Newton, MA 02458, USA; 3 Department of Sociology, Brown University, Box 1916, Providence, RI 02912-1916, USA; 4 Center for Environmental Studies, Brown University, Box 1943, Providence, RI 02912-1943, USA; 5 Communities for a Better Environment, 1440 Broadway Suite 701, Oakland, CA 94612, USA

## Abstract

**Background:**

Exposure assessment has shifted from pollutant monitoring in air, soil, and water toward personal exposure measurements and biomonitoring. This trend along with the paucity of health effect data for many of the pollutants studied raise ethical and scientific challenges for reporting results to study participants.

**Methods:**

We interviewed 26 individuals involved in biomonitoring studies, including academic scientists, scientists from environmental advocacy organizations, IRB officials, and study participants; observed meetings where stakeholders discussed these issues; and reviewed the relevant literature to assess emerging ethical, scientific, and policy debates about personal exposure assessment and biomonitoring, including public demand for information on the human health effects of chemical body burdens.

**Results:**

We identify three frameworks for report-back in personal exposure studies: clinical ethics; community-based participatory research; and citizen science 'data judo.' The first approach emphasizes reporting results only when the health significance of exposures is known, while the latter two represent new communication strategies where study participants play a role in interpreting, disseminating, and leveraging results to promote community health. We identify five critical areas to consider in planning future biomonitoring studies.

**Conclusion:**

Public deliberation about communication in personal exposure assessment research suggests that new forms of community-based research ethics and participatory scientific practice are emerging.

## Background

Tired of government inaction toward community concerns about pollution from refineries in her neighborhood, Ethel Dotson, a 53-year resident of Richmond, California, decided it was time to up the ante: armed with 10 vials of her own blood, she and several other residents gathered in front of California's Hazardous Materials Laboratory and demanded that officials test their blood for dioxin and other contaminants. "I have a right to know what's in my body," she argued [1]. Dotson's demand to document 'chemical trespass' [2] in her body reveals the scientific promise as well as policy and ethical challenges of the rapidly expanding field of chemical biomonitoring.

Biomonitoring, or body burden research, involves the assessment of the presence and concentration of chemicals in humans by measuring the parent chemical, its metabolite, or reaction product in human blood, urine, breast milk, saliva, breath, hair, or other tissue [3]. Biomonitoring as a tool for human exposure assessment has been used for decades, particularly in occupational settings [4,5], and, perhaps most commonly, for lead, starting in the late 1800s [6]. Later, biomonitoring studies by the Centers for Disease Control (CDC) during 1976–1980 documented significant declines in blood lead levels corresponding to the decline of lead in gasoline [7]. Population-based biomonitoring of blood lead levels in children was adopted to track the effectiveness of poisoning prevention strategies and to detect cases where exposures from homes contaminated with lead paint need to be remediated [8].

As public health increasingly targets the environmental determinants of chronic diseases, biomonitoring is fast becoming a key strategy for providing a scientific basis for prevention via exposure reduction and motivating action. These efforts rest on newly developed analytical methods that detect ever lower concentrations of an increasing number of chemicals for which animal and cell studies show troubling biological effects, but human exposure levels, exposure sources, health effects, and exposure reduction strategies are not yet well understood. As stated in the 2006 National Academy of Sciences' report *Human Biomonitoring for Environmental Chemicals*, although biomonitoring has advanced significantly, researchers, regulators, and decision-makers face new challenges about how to interpret, report, and act on results that only partially elucidate links between environmental chemicals and health [9]. This paper heeds NAS's call for additional research that elucidates new approaches for addressing the scientific and ethical challenges of biomonitoring results communication in the United States. Although we focus on exposure biomonitoring throughout, parallel issues are raised by other personal exposure assessment methods, such as dust and air samples taken from an individual's home.

Exposure assessment has always been one of most methodologically challenging aspects of environmental health science and environmental epidemiology. The last ten years have seen significant advances that allow scientists to assess and characterize chemical body burdens and potential health risks, including those that may have more than one route of entry into the body (e.g. inhalation, ingestion, and dermal) [10,11]. Biomonitoring techniques can be divided into three basic categories: biomarkers of exposure, effect and susceptibility [12,13]. Previously restricted to logistically challenging and costly academic research and occupational cohort studies, biomonitoring techniques, particularly for exposure, have become more widely available, practical, and less expensive. This has resulted in the proliferation of biomonitoring studies among scientists in academia, state and federal agencies, environmental advocacy organizations, and non-profit research institutes.

The visibility and policy impacts of this new wave of biomonitoring have been profound. Scientific journals and the media have reported on a flood of studies, including flame retardants in breast milk [14,15], pesticides in umbilical cord blood [16], and endocrine disrupting compounds (EDCs) in household air and dust and householders' blood and urine [11]. In 1999, the federal government began systematically tracking personal exposures in a representative sample of the US population [17]. When results were released in April 2001, Richard Jackson, then-director of CDC's National Center for Environmental Health, predicted that biomonitoring "could be revolutionary for environmental health research in the United States" [18], as these new exposure assessment methods and the data they provide are much-needed to advance environmental epidemiology, environmental health policy, and regulation. Recent CDC biomonitoring results have highlighted the effectiveness of antismoking efforts, including banning smoking in public places, and have focused attention on surprising exposures of women of reproductive age to hormonally active chemicals from consumer products [19]. CDC has continued issuing body burden reports every two years, expanding the number of chemicals tested, and is encouraging states to develop their own biomonitoring programs [7,19,20]. In September 2006, California became the first state to do so [21].

Although biomonitoring is a direct indicator of human exposure to certain compounds and their metabolites, this technique cannot generally be used to easily identify their sources. As one biomonitoring study participant states: "None of these chemicals come with a return address." Moreover, these techniques are rarely able to predict health outcomes or even sub-clinical effects in humans. As biomonitoring procedures become less costly, researchers have expanded the array of chemicals being studied, yet many of these substances lack toxicological or epidemiological evidence regarding their potential health effects [22] and regulatory benchmarks for comparison [23]. This makes it imperative to address some specific ethical challenges of biomonitoring.

We first review the evolution of exposure assessment science and the emerging ethical issues associated with the proliferation of biomonitoring techniques and the communication of results. We then describe our methods for recruiting and interviewing individuals involved in biomonitoring research about their opinions and practices. Our results identify three approaches used by academic scientists and environmental organizations to communicate information about the effects of chemical exposures on health and to leverage regulatory and policy change: 1) clinical ethics; 2) community-based participatory research; and 3) citizen science 'data judo.' The first is biomedically driven, while the latter two emphasize prevention research and advocacy. Our results also reveal the ethical and administrative issues faced by scientists when considering whether and how to report individual exposure information to study participants. We then report on guidance offered by government publications, professional association "best practice" guidelines, and journal articles concerning individual results communication and make recommendations based on our interviews with researchers currently collecting and reporting individual exposure data. We conclude with some of the ethical considerations for future work in this area.

### Evolution of Exposure Assessment Science and Emerging Ethical Issues

There is little guidance for scientists and academic-community research collaboratives that want to report individual and community-level exposure data to study participants. One of the first fields of public health to grapple with the issue of biomonitoring and individual report-back was occupational health. In the 1960s, testing of blood, urine, and other tissues was a well-established practice for occupational health surveillance and research, although only a small portion of the many chemicals common in work environments were consistently examined, in part due to limitations in chemical analytic capacity [19,24]. Occupational health researchers typically conduct retrospective occupational cohort studies, in which morbidity and mortality records for a population of individuals who worked in a particular environment are analyzed to determine associations between exposures and adverse health outcomes. Historically, cohort members were not notified about individual findings, even though this information could have served as a basis for efforts to reduce exposures or conduct health screenings to potentially reduce morbidity and mortality risks. However, in a speech delivered to NIOSH in 1982, bioethicist John Fletcher called on epidemiologists to "join other biomedical scientists who have the obligation to notify study subjects" [25]. The notification of individuals in a cohort study is now standard [25-27], and is explicated in OSHA's Hazard Communication Standards of 1983 and 1987 [26,27].

Technical developments now enable environmental health researchers to widen their gaze from a previous focus on pollutants in occupational settings and contaminated environmental media such as air or water toward contaminants in human tissues. Moreover, smaller-scale biomonitoring studies conducted by environmental advocacy organizations have been effective vehicles for promoting precautionary approaches to chemical regulation [7,17,28]. For example, policymakers in Europe and California used data from breast milk monitoring to encourage a recent phase-out of certain PBDEs (polybrominated diphenyl ethers), flame retardants ubiquitously used in electronic equipment, furniture, and other products [29]. This strategy of advocacy biomonitoring has been replicated by numerous organizations in the U.S. and abroad to address other potential hazards, such as parabens in cosmetics and exposures from PFOA (perfluorooctanoate), which is used to manufacture Teflon [30].

New ethical dilemmas have emerged regarding the reporting of exposure data, especially since information about health outcomes and dose-response relationships is uncertain or not available [31,32]. Indeed, nearly 85,000 chemicals are currently registered for commercial use, yet barely ten percent have undergone basic toxicity testing, and these do not include assessments of carcinogenic, developmental, reproductive, neurological, immunological, or endocrine effects [22]. Quantifying chemicals in biological samples inherently precedes understanding their effects, because exposure measurements are needed before we can evaluate links to health [4,33]. Thus, scientists are confronted by the question of whether an ethical obligation exists to notify participants of their exposure results, or to withhold this information if it does not offer clear insights on health effects or the sources and pathways of exposure. Moreover, the implications of results for individuals and communities are further complicated by interactions of environmental exposures with individual and population differences in genetics, nutrition, health status, health-related behaviors, and lifetime exposures to other contaminants [34].

Few precedents exist for reporting biomonitoring data to individuals when there is little information for interpreting health implications. The first discussion of this issue was the Department of Health, Education and Welfare's 1979 Belmont Report [35]. The report's guidelines for protecting human subjects in research rest on four principles: *autonomy*, which includes the right-to-know (or the right-not-to-know) as a basis for self-determination in acting on research results; *beneficence *and *non-malfeasance*, which together encompass the researcher's responsibility to maximize good and minimize harm; and *justice*, which refers to the distribution of benefits and harm [35]. "Autonomy" and "justice" weigh in favor of reporting individual results to study participants. "Beneficence" encourages researchers to consider benefits, such as empowering individuals and communities to take actions to direct their clinical care, reduce hazardous exposures, protect their health, and participate more fully in public health research and policy. "Non-malfeasance" considers the potential for report-back to result in fear, worry, stigma, or legal and economic complications (related to health insurance or property values, for example) and the possible promotion of unnecessary or counter-productive interventions [30]. Although public health professionals have developed methods for reporting to individuals on regulated contaminants such as lead, study participants often are not informed of their personal results that lack regulatory or clinical significance. The issue of reporting individual-specific data to participants has traditionally been more of a concern in clinical medicine since public health studies generally have dealt with community-level data, such as cancer registry information or environmental contamination data in media such as food and water [27].

Finally, biomonitoring has implications for environmental justice. Communities that are socially, economically, and politically marginalized – from Native American communities in Akwesasne, New York, and St. Lawrence Island, Alaska, to African-American communities in Anniston, Alabama, and New Orleans – are beginning to conduct biomonitoring research to track exposures (cross-sectionally and longitudinally), record the extent of community-specific contamination, and leverage government funding, industry action, or legal remedies. However, environmental justice advocates have approached biomonitoring with caution because of concerns that "after-the-fact" measurements cast communities as environmental hazard detectors [36]. Furthermore, this strategy can potentially "over-scientize" environmental health problems, overlooking upstream causes rooted in social inequality, economic exploitation, and racial discrimination [37,38].

As a powerful and scientifically contested method, elucidating the ethical and policy implications of biomonitoring is critical for providing guidance to those who design biomonitoring programs and for those faced with the daunting task of interpreting uncertain data and making decisions about how to protect health.

## Methods

Our interest in this area stems from our own research that entails environmental sampling of household air and dust, as well as biomonitoring, to assess the presence of endocrine-disrupting chemicals potentially linked to breast cancer [30]. The research partners in this project have chosen to report aggregate exposure assessment results through peer-reviewed publications, media outreach, and public meetings, and to report individual results back to study participants. As we developed this project, we wanted to determine what information on report-back protocols is currently available to researchers and communities. This included examining exposure reports published by government agencies, "best practice" guidelines issued by professional associations, and journal articles on individual studies. We also interviewed scientists and community members involved in biomonitoring research. We specifically assessed how documents presented exposure data, and what information, if any, was provided about interpreting and acting on the exposure data. We also examined how scientific uncertainty and data gaps were explained to communities and study participants. We interviewed other scientists doing exposure studies to see how they decided on report-back processes. Interview protocols were reviewed and approved by Brown University's Institutional Review Board. We began by contacting colleagues who were involved in academic and advocacy biomonitoring research, and added to our sample researchers that our colleagues recommended, through snowball sampling. Our data come from 26 interviews, a review of relevant literature, and participant-observation at conferences and workshops where these report-back issues have been debated and discussed. Un-cited quotations and information come from those interviews and observations.

## Results

### Frameworks for Communicating Biomonitoring Results

When considering the issue of whether and how to report individual data to study participants, scientists must weigh participants' right-to-know and the potential benefits of receiving the information against the possible psychological or financial harm of trying to make sense of data that may not provide a clear picture of potential health implications or guidance on how to reduce exposure [25,26]. Our interviews, observations, and assessments of the literature revealed three distinct frameworks used by scientists for reporting back biomonitoring results: 1) *clinical ethics*, a biomedically-driven approach; 2) *community-based participatory research (CBPR); *an approach focused on prevention research and 3) *citizen-science data judo *an advocacy-driven approach. The distinctions between these frameworks are summarized in Table 1.

**Table 1:** Frameworks for Communicating Biomonitoring Results

Framework	Orientation	Right-to-Know Emphasis	Communication Strategy	Protocol Development
***Clinical Ethics***	Biomedical	Weak	Individual results communicated if exposures reach clinical action levels, or if exposure/health outcome relationships are understood.	Protocols developed primarily by scientific and medical experts. Participant confidentiality is paramount. No opportunities for participants to share results with each other.

***Community-Based Participatory Research***	Prevention	Strong, while also protecting participants' right-not-to-know their results.	Encourages communication of aggregate and individual-level results to study participants with an emphasis on explaining scientific uncertainties and addressing concerns about community stigmatization. Participant right-to-know explained at the point of study recruitment and consent.	Protocols developed jointly by scientific and community partners. Confidentiality of participants is important, although some studies may offer opportunities for participants to share results with each other, if they wish. Protocols seek to balance community-right-to-know with individual right-to-know.

***Citizen-Science Data Judo***	Advocacy	Strong	Encourages report-back of aggregate and individual-level results to study participant to support precautionary individual action, communications, and policy change.	Protocols developed by scientific experts affiliated with advocacy organizations, sometimes with consultation from study participants. Participants encouraged to share results with each other and to speak publicly about their results to the media and broader public.

1) *Clinical ethics *assumes that decisions about whether and how to report individual biomonitoring results rests with scientists and medical experts, and should be based on whether the risk relationship between exposure and health effects is understood [39]. For biomarker levels when an exposure-health outcome relationship is known, the clinical action level, or "the level at which biomarker results will be of concern," should be determined prior to the start of the study [40]. If the results fall below this clinical action level, individual data is generally not reported to participants. The clinical medicine model gives more weight to the expert-researcher's role in avoiding possible harm to study participants from reporting uncertain information and less weight to the study participants' ability to process complex and uncertain scientific information and respond autonomously. The clinical ethics approach may preclude precautionary action by participants whose biomonitoring results may approach but still be below an "action level," or regulatory benchmark of concern, even if the evidence suggests that there are health effects below the action level, as in case of lead or mercury. Moreover, the clinical ethics framework offers a narrow view of the potential for beneficial action – usually focused on medical intervention or public health interventions based on regulatory guidelines or a legal mandate (such as child lead screening). In certain instances, these regulatory benchmarks are legally or scientifically contested. In practice, the clinical ethics framework overlooks the significant evolution of clinical communications. In particular, patients have become more proactive in directing their own health care, often by tracking screening results, such as blood pressure and cholesterol, even when levels fall below a clinical action criterion [39,41]. In addition, the potential for individual-level data to provide relevant information on an individual's health is further complicated by the possibility of future scientific advancements in establishing links between exposure and health outcomes. Indeed, as one academic research scientist interviewed stated:

" [individual results] are part of their medical history, so potentially in a few years that might be useful information."

Because no health effects are conclusively linked to individual low-level exposure for the majority of chemicals tested in biomonitoring studies, this clinical framework will likely lead researchers to report data only on an aggregate level. Nevertheless our interviews with three medical doctors conducting biomonitoring research suggest a potential shift in the clinical ethics framework. Based on their experiences as practitioners and researchers they saw certain advantages of engaging participants openly about biomonitoring results and their uncertainties to ensure productive clinical interactions.

While health-based benchmarks are unavailable for most of the chemicals tested in humans, population surveillance biomonitoring programs that have emerged over the last ten years provide useful comparison data for individual biomonitoring results. Indeed, scientists involved in an epidemiological cohort study of the developmental effects of pesticide exposures explained that the research team began by only reporting aggregate biomonitoring results, but subsequently reported individual-level results because exposure levels could be compared to the national average levels provided by the CDC's biomonitoring information [19]. Although these comparisons are useful, they often do not help elucidate potential exposure pathways and sources nor do they relate exposures to levels that have been associated with health effects.

2) *Community-based participatory research (CBPR*) is a framework in which decisions about individual results communication rest equally between scientists and the study community. This approach assumes that individual and aggregate-level reporting of study results can empower communities and individuals to act on scientific evidence [42] and can restructure unequal and discriminatory power relationships [43]. The approach stipulates that the sharing of knowledge (such as biomonitoring results) between researchers and participants can have an impact beyond the relevance of the knowledge for individual health [44]. Therefore, CBPR encourages as much information dissemination as possible to study participants, and posits that ownership of collected data lies primarily with the individual participants from whose homes or bodies the original samples were taken [42].

A recent article published on reporting pesticide exposure results to farm worker families in North Carolina echoes this approach, stating that, "Communicating risk to affected individuals should be an integral part of any community-based project. It is ethical to return information to the owner of that information" [45]. Indeed, investigators in this farmworker study assumed that individual report-back for all chemicals analyzed would occur, and therefore the main question was not *whether *to report individual results, but *how*. According to this CBPR framework, even information about an exposure for which a corresponding risk relationship is not available can have some benefits to participants, such as taking action to reduce personal exposures. The North Carolina study emphasizes community involvement in the development of report-back protocols to address the interests and concerns of study participants:

*In terms of the ambiguity, [the participants] thought it was important that the scientists present la verdad (the truth). If this meant telling women that it was not possible to know the level of danger represented by the findings, they would prefer to know that rather than to have the scientists give them a simpler but incomplete answer *[45].

Thus, the CBPR approach to reporting data assumes that results should be disseminated to participants not only to communicate health information, but also to address disparities in access to knowledge that traditionally characterize 'lay-expert' relationships [46]. The CBPR approach must be strategic, however, since this framework raises potential conflicts of community versus individual right-to-know: the broad dissemination of biomonitoring results can adversely affect communities under study, even if the rights and confidentiality of individual study participants are protected. Indeed, communities exposed to toxic contaminants with significant health risks, may be collectively or individually stigmatized. Individually, they may be denied jobs, health or life insurance if they are associated with an "at risk" population. Collectively, a community perceived as "contaminated" may be passed over for programs or benefits, face stereotyping that affects the quality of health care, or suffer lost real estate values or financial liability for remediation [47]. For example, as early news broke of elevated PCB levels in the community of Broughton Island in northern Canada, and before the full extent of contamination was understood to extend throughout the circumpolar region, Broughton Islanders were initially shunned as the "PCB people" with an adverse impact on the livelihood of the fishing community [[48], p. 108]. These potential pitfalls of report-back and right-to-know can be proactively addressed if researchers purposefully develop protocols and communication strategies in partnership with study communities of a biomonitoring project [30,49]. Key to this process is a collective understanding about who represents the interests of study communities and how their issues can be effectively deliberated and incorporated into protocol development.

*3) Citizen science 'data judo,' *or what we term "advocacy biomonitoring," is a strategy in which study design and individual results communication are shaped primarily by policy goals to improve chemical regulation. Indeed this framework assumes that personalized information about chemical body burden can broaden public support for toxics use reduction policies, and motivate individuals to pursue both collective activism and individual exposure reduction. Environmental advocacy groups and communities marshal their own scientific resources and expertise to conduct research, and report-back strategies are specifically aimed to advance regulatory and policy change [50]. Our interviews with scientists who conducted biomonitoring studies for environmental organizations, as well as the individuals who participated, support this framework.

Although the 'data judo' approach to report-back has overlapping goals with the CBPR framework, there are some important differences. While CBPR is primarily research driven and aims to use report-back strategies in order to break down power and knowledge disparities between scientists and communities, the data judo approach is advocacy-driven and explicitly seeks to mobilize constituencies by increasing public awareness about a specific regulatory issue or policy initiative. Over the past five years, there has been a proliferation of body burden studies spearheaded by environmental organizations. Three milestone activist body burden studies were conducted by the Environmental Working Group (EWG). The first study, known as the *Body Burden Study*, recruited nine volunteers, most of whom were prominent environmental advocates, to have their blood and urine tested for the presence of 210 chemicals commonly found in consumer products and industrial pollution streams [28]. An average of 91 industrial compounds, pollutants, and other chemicals were found in the blood and/or urine of the study participants, with a total of 167 chemicals found in the entire group. The report on this study appears on the EWG website where viewers can click on a thumbnail photo of each study participant to see what contaminants are in that person's body.

The second EWG study examined the presence of a category of brominated flame retardants (PBDEs) in the breast milk of 20 first-time US mothers [29]. This study found an average level of bromine-based chemicals in breast milk that was 75 times the average found in recent European studies [51,52]. Milk from two study participants contained the highest levels of fire retardants ever reported in the United States, and milk from several of the mothers in EWG's study had among the highest levels of these chemicals yet detected worldwide. The third study examined the presence of chemicals commonly used in cosmetics and body care products in teenaged girls. The study detected sixteen chemicals from four chemical families – phthalates, triclosan, parabens, and musks – in blood and urine samples from twenty participants aged 14–19 years old. Many of these chemicals are linked to potential health effects, including cancer and hormone disruption [53].

Advocacy biomonitoring has made the image of ubiquitous human exposures to chemicals resonate widely in the media, regulatory, and policy arenas, and has led to a proliferation of studies by several other environmental organizations and media outlets, including Commonweal, World Wildlife Federation, Greenpeace, Environmental Defence (Canada), the Sightline Institute, National Geographic, and a major newspaper in Oakland, California [15,54-59]. Advocacy biomonitoring highlights the failure of environmental regulations and policies, such as the Toxics Substances Control Act (TSCA), to protect the public from exposures to ubiquitous contaminants, most of which have not been tested to assess their potential short- and long-term health impacts. Many of these studies also raise questions about whether current regulations are effective at protecting environmental health. As a result of extensive public outreach by both organization scientists and study participants, advocacy biomonitoring has garnered substantial regulatory attention, and legitimated mounting public concern about the ubiquitous presence of these chemicals in consumer products and diverse environments [60]. With few exceptions, these advocacy studies report data to study participants individually and also provide opportunities for them to talk publicly about their results. For example, EWG provides online personal biographies of study participants in their Body Burden and Breast Milk studies [28,29,61]. Many of these biographies emphasize participants' efforts to lead 'healthy lifestyles' and the fact that they did not work directly with chemicals in their jobs or live near major pollution sources. Participants in advocacy biomonitoring studies savored the opportunity to share their results with other study participants to better contextualize their meaning and highlight opportunities for exposure reduction. As one participant noted:

...*the important thing, I think, to me, was understanding my results in the context of other people's results. So that while each of us got our results individually... it was only sort of when most of us [study participants] agreed to be in a conference call together to talk about it that I sort of began to understand what my own results meant, and how I felt about it in the context of other people's reactions... And so it was very important to me that as a group we agreed to share our results. Not that we now know exactly what it means, but it was interesting to note that the biggest fish eaters had the highest levels of mercury*.

One of the more controversial aspects of advocacy biomonitoring is that it explicitly challenges traditional Institutional Review Board (IRB) protocols of protecting participant confidentiality, by giving study participants opportunities to discuss their results publicly, with the media and each other. Based on our interviews with academic scientists, many IRBs have traditionally allowed aggregate reporting of study results, while restricting or strongly discouraging the conveyance of individual-level information. For example, some academic IRBs require passive individual report-back protocols, which prohibit researchers from asking participants if they want to receive results. Although IRB concern about participant confidentiality is warranted, report-back protocols that require greater initiative on the part of study participants to acquire their results ignores the fact that many individuals want their own data in order to take individual or collective action to reduce exposures. Participants may also want to share their personal results with other study participants or collectively through their own networks, communities, and public forums. As one scientist from an advocacy organization argued:

*I think part of the challenge for all of the biomonitoring studies that are going on, including ours, is that you want to do it by the book, so that you write up an IRB [protocol] like any other study with human subjects, but in a way, doing it by the book is exactly what this is not about*.

Therefore, some advocacy biomonitoring studies have encouraged IRBs to examine how traditional standards of confidentiality may impose problematic restrictions on individual results communication. For some communities, these restrictions can be perceived as undermining the capacity of study participants to understand the implications of the study and to take protective action by first comparing their individual results in the context of those of their peers.

The above analysis of these three approaches to biomonitoring results communication also elucidated some general guidelines for reporting exposure data to study participants.

### Central Issues in Reporting Exposure Data to Individuals and Communities

#### 1) Providing background information to make individual results meaningful

Several scientists and participants recommended comparing individual data with aggregate study results. Such comparisons are useful for placing the information into a familiar context. As Quandt et al. found: "presenting individual exposure data with reference to actual community data, rather than more abstract population-level reference data, engages community members' interest." [45]. The use of comparisons is also recognized in the literature on risk communication as important when the values being communicated appear small, or when risks are unfamiliar to the involved community [62]. Body burden studies can fit both of these criteria: chemicals are often detected in seemingly low concentrations and they may involve chemicals unfamiliar to the general population.

Another system for reporting individual-level data is to compare it with other published studies, such as the CDC reports [17,19,20], when such studies exist. It is important to keep in mind, however, that there can be some confusion about what this comparison implies. For example, one researcher indicated that when pesticide exposure results were reported to individuals, it was critical to ensure that any comparisons to general population levels from the CDC report were not misinterpreted as safety benchmarks. In this way, the exposure distribution for the population often stands in as a substitute 'population norm' [40,45]. This can have two potential negative effects on the participants' understanding of their risk: (1) it can lead to a false sense of security, with participants who have exposure levels at or below a community average and (2) it can lead to unnecessary concern when those with higher exposure levels than the study average assume that they have unsafe levels, regardless of the fact that the entire cohort might have exposures that are significantly below levels that indicate cause for concern. One scientist we interviewed, who directed an exposure study on brominated flame retardants, indicated that two study participants had extremely high levels of PBDEs in their tissue samples. This caused at least one participant to be concerned, although currently there are no human health studies to indicate whether or not her results posed health risks for her or her child:

*The participant who had the second-highest result was really pretty blown away by it. She had done the study expecting that she would be one of the more healthy, safe, you know, protected... It's really an unfortunate part about enrolling [participants] in studies and giving them results about contaminant levels in their bodies when you don't have an even distribution or a way that would kind of predict or prepare them for where they might be in that distribution and she took it really hard ... the rest of the ... [participants] felt lucky and felt protected*.

It is clear that using study or population exposure distributions as a way to interpret individual-level data has potential pitfalls. However, this fact should not prevent exposure distributions from being reported in the context of individual-level results. Instead, care should be taken to ensure that study participants understand that population averages should not be considered safety benchmarks. Whenever possible, information about comparison measures in other populations should be coupled with an explanation of the potential health implications of these levels and appropriate regulatory benchmarks.

#### 2) Developing report-back protocols and contention among researchers regarding individual versus aggregate communication of results

Our interviews revealed that the process for developing report-back protocols varies widely, both among academic and advocacy biomonitoring studies. Some researchers develop report-back protocols with little community input, while others solicit input from the study community, scientific colleagues not directly involved in the study, and social scientists. Most interviewees acknowledged the importance of having community representatives involved in the decision of whether and how to report individual and aggregate study results. They felt that it should be the community's decision whether individuals receive their own data, especially in situations when studies included participants whose illness was potentially linked to a substance under study. Nevertheless, for academic studies or research involving community-academic collaborations, this sentiment must contend with the fact that all entities that receive federal funding for research must operate in accordance with federally prescribed IRB procedures; this makes IRBs the final arbiters of whether or not to approve individual-level notification of study participants about biomonitoring results. The academic scientists we interviewed reported a wide variation in the willingness of their IRBs to grapple with the bureaucratic and logistical challenges of reviewing and approving individual-level report-back protocols for biomonitoring studies. In addition, some described a lack of consensus among study collaborators, including academic scientists and members of community advisory panels, about whether to report individual data. The disagreements over how to design report-back protocols show that, even when there is a commitment to right-to-know and community-based research, deciding how to report to individuals in biomonitoring and exposure assessment studies may not be simple to negotiate among collaborators. For example, physicians sitting on an advisory board for one biomonitoring study tended to discourage individual report-back due to concerns that patients may have health-related questions linked to their study results that most doctors could not realistically answer. Conversely, community advocates and some industry representatives tended to favor releasing individual results to study participants, viewing this as a right-to-know issue.

#### 3) Factors affecting how results are reported

Most scientists described a system of individual and aggregate report-back that involved a combination of written materials and conversations with experts, either over the phone or in person. Some had a form of passive reporting, where study participants could contact researchers if they wanted to confidentially receive their personal results. This system also gives participants the opportunity to opt out of receiving their individual-level information. Another researcher stressed the need to follow up report-back with support from a counselor and/or to have someone participants can contact down the line when questions arise related to emerging health issues or new concerns. One scientist discussed the need to remain extremely flexible and available for participants, since a third of the participants who did not opt to call in for results later expressed interest in getting their results during a follow-up survey a few weeks later. This suggests that passive reporting may not be sufficient for providing results to participants who want them.

The report-back process offers the potential to use aggregate and individual-level information to develop exposure reduction interventions. Indeed, receiving information about how to remove pesticides from the home or how to prevent future contamination was reported by participants to be the most important part of the report-back process in two pesticide exposure studies. The promotion of public health interventions directly related to study results helps scientists ensure that the information provided to participants has a positive effect on their ability to take action to promote health and well-being [63]. One scientist brought up the importance of reporting individual study results in combination with specific exposure reduction recommendations that participants can follow individually:

*The most important component of that for us was not only giving the information but giving information about what the women could do. So that reporting back is always linked to action, so that they are not getting the information without having any idea of what they can do about it*.

In one pesticide study, the health workers explained direct actions that all women could take to prevent pesticides from entering their homes and getting picked up by children, including closing windows during crop-spraying, and having farm workers change clothes before entering the home. In addition, brochures were provided, with information in Spanish about storing and washing work clothes separately and the idea of pesticide residues being invisible [40,45]. Other biomonitoring studies of persistent organic pollutants that bioaccumulate up the food chain provide participants with information about how to reduce their consumption of animal products or decrease the presence of contaminants in household dust by switching to less toxic consumer products. However, scientists are often forced to balance the potential disruption and cost of an intervention with the strength of the information indicating a pollutant's origins and health impacts [30]. For example, one scientist leading a study on brominated flame retardants indicated that he would provide participants information on how to reduce levels of animal fat in their diet, citing other health benefits associated with this action. On the other hand, he also indicated that he would refrain from advising participants to take more costly or inconvenient action to minimize the presence of PBDEs in household dust. This is especially true if the effectiveness of these interventions has not been assessed:

Right now my gut feeling would be not to tell people you should throw away all your furniture and buy all new furniture. That seems kind of extreme, right?

Thus, it seems that in the case of PBDEs, for which the health effects are less well understood, the decision of whether or not to provide suggestions for exposure reduction involved balancing the level of confidence in the efficacy of the possible intervention with the disruption that the intervention would cause, and whether or not the intervention has other public health benefits besides minimizing pollutant exposures, such as reducing animal fat content in the diet, which can reduce the risk of heart disease.

Some biomonitoring results raise conflicts with an existing public health practice with a known health benefit, as with breastfeeding. While there are indications that PBDEs may have potential developmental health effects on offspring, toxicological evidence suggests that most of these effects occur *in utero *rather than through exposures through breastfeeding [14]. However, breast milk studies have been controversial because of concerns that they may discourage breastfeeding, despite its known health benefits. A recent survey of breastfeeding women suggests that learning about the presence of chemicals in their breast milk may lead them to wean earlier than intended [64], although the survey for this study was hypothetical and did not actually measure whether in fact reporting monitoring results actually changed the duration of breastfeeding. Although further research is needed to examine whether in fact reporting biomonitoring results actually changes breastfeeding behaviors, this issue remains controversial among public health advocates. To respond to this debate, a recent article proposed a model informed consent protocol for breast milk biomonitoring studies that includes "advice that breastfeeding is almost always considered to be the best form of nutrition for a baby, and that the fact that the study is being carried out should in no way be taken as implying anything to the contrary" [65]. All three scientists we interviewed who were involved in breast milk studies reported that they encouraged participants to breastfeed. Empirical investigation of mothers' responses in breast milk studies that encourage breastfeeding could inform the design of future monitoring efforts.

Finally, debates over "risk messaging" related to biomonitoring research are most difficult when health implications warrant exposure-reduction, but interventions are either impossible, unjust, or would produce more deleterious consequences. In the 1980s and early 1990s, communication of biomonitoring results among Arctic Inuit communities called into question the consumption of their traditional food source of large marine mammals. Contaminants bioaccumulate and are delivered through many marine mammal food sources that are essential to community survival, subsistence and hunting culture. In this context, the conundrum lies in the paucity of viable alternative foods sources. Imported, market-based foods pose their own, arguably more dire health consequences in the form of malnutrition, obesity, cardiovascular disease and diabetes [49,66]. Increasingly, messages encourage consumption of particular species with lower contaminant levels or specific cuts of flesh. Yet, mounting evidence of the reproductive, immunological, and developmental effects of these persistent contaminants leave many communities and scientists in an uncertain situation in terms of report-back strategies [49]. Scientists and community members involved in these studies support community right-to-know; however, this work also poses a significant challenge since exposure reduction strategies are extremely difficult to employ. In the case of the Inuit, efforts to reduce pollutant levels in marine mammals require international political action and a long time horizon, given the environmental persistence of some contaminants [66,67].

#### 4) Addressing varying levels of literacy

Biomonitoring studies involve populations with varying levels of literacy [68,69]. In some cases, as with the EWG breast milk study, participants are pooled from populations of environmental activists who already have high levels of environmental health literacy. One academic scientist we interviewed who was conducting breast milk biomonitoring, noted that participants came from two distinct groups, one that was upper middle class with a post-graduate level of education, and another that was working class, with a high school or lower level of educational attainment. The latter group was far less inclined to seek their biomonitoring results. However, participants who are members of marginalized groups with low levels of scientific literacy may be eager to hear their results with a preference to have materials read to them and be shown diagrams, graphs and pictures to interpret data [45].

Another scientist involved in a cohort study on pesticides in low-income urban women and children provided further evidence that populations of low literacy are interested and can demonstrate a high level of comprehension in interpreting individual results:

Yeah, the research workers have been getting the same questions that they've been getting for years now, you know, when are we going to get our individual results for our kids? You know when are we going to know about pesticides? When are we going to know the results from our [monitoring] ...?

Thus, successfully conveying complex results to populations with low levels of scientific literacy requires carefully crafting report-back protocols so that participants are engaged and able to understand the material presented to them. It is also necessary to communicate with members of the participant community during the creation of report-back materials, to ensure that the information is relevant to their life experiences. If these measures are successfully undertaken, our interviews with researchers suggest that populations with lower levels of scientific literacy are as interested in receiving their individual data as more educated groups are. Ultimately, a participant's decision about receiving individual results is a personal one, and researchers must ensure that participants can make a clear, deliberative choice regarding their right-to-know or not-to-know.

#### 5) IRB requirements, standards of confidentiality, and individual-report-back

Although IRBs focus on protecting the rights and confidentiality of individuals, this may not require that individual results be reported back to study subjects. In fact, under certain circumstances, IRBs may discourage individual report-back. The scientists we interviewed faced a range of responses from IRBs to their report-back protocols. One researcher recounted how the IRB initially opposed releasing individual study results to participants. However, he was able to convince IRB members to reconsider their decision by demonstrating that community representatives on the study's advisory board supported the report-back protocol. Another IRB limited researchers to calling participants and referring to them by their individual code number, rather than their names in order to protect confidentiality. Conversely, environmental advocacy organizations that conducted studies gave participants numerous opportunities to discuss their individual results with each other. In one study we examined, conference calls were held for all participants before and after results were disseminated and participants were encouraged to share their personal response to receiving their results with the group. The benefit to participants was that this process enabled them to share thoughts regarding pollutant sources and ways to reduce exposures. This approach encourages a reevaluation of traditional protocols aimed at protecting participant confidentiality and suggests new ways for researchers to enhance the participatory nature of disseminating and interpreting biomonitoring results. An IRB's duty to protect confidentiality ensures that personal information is not released without a participant's explicit desire and instruction. Nevertheless, as with any health information, a person should be free to share their information with others, as long as they do not consciously violate other people's desire to not share their data.

## Discussion

Biomonitoring provides new techniques and innovation in environmental health science for detecting and understanding the health implications of chemical trespass in people's bodies. Some biomonitoring projects are done by academic, government, and regulatory institutions and involve varying degrees of lay involvement. Others are done by environmental health advocacy organizations in order to mobilize the public and lobby relevant officials and legislators for regulatory and policy change. Biomonitoring also raises new ethical challenges that require democratizing the research enterprise to allow study participants to play a larger role in interpreting, disseminating and leveraging study results to take action.

A consensus has yet to emerge regarding the ethics of reporting individual data on environmental exposures when the relationship between exposures and health outcomes is not established [70]. Indeed, some environmental health advocates and scientists who generally support the notion of community right-to-know remain wary of individual notification of data when the clinical implications are uncertain. For example, recent studies on the presence of PBDEs, PCBs, and other toxins in breast milk have raised some controversy about how to report biomonitoring results in light of the many known benefits of breastfeeding [71]. Nevertheless, participatory research models are spilling over into the environmental health arena, compelling more scientists and advocacy organizations to think through the issue of whether and how to provide individual-level biomonitoring information [30,45]. The literature and our interviews with scientists and study participants conducted for this study, although not unequivocal, indicate a trend in favor of addressing report-back strategies in the recruitment and consent process for research studies. Our research suggests that the ethical issues of reporting back exposure monitoring results necessitates addressing the rights of study participants to information before, during, and after studies so that they can make informed decisions and be empowered to take action. Study participants often want their individual results and an interpretation of them in terms of what potential exposures may mean for their health or opportunities to reduce exposure. However, researchers and public health practitioners face ethical issues in interpreting exposure results when health and safety data are not available for the pollutants under study or when there is no scientific consensus about the risks associated with exposures or efficacy of exposure reduction strategies.

Perhaps the most important issue to emerge from our interviews with academics, scientists from advocacy organizations, and study participants is that it is desirable to set expectations for any exposure assessment or biomonitoring study *before *commencing data collection and setting up results communication protocols. One important aspect of this effort is to clarify the inherent scientific limitations of interpreting what the data collected could mean for community and individual-level health. Equally important, even if health implications are unknown, individual-level report-back can provide an impetus for people to take individual action that could reduce their exposures. It can also provide participants with opportunities to collectively leverage results to support advocacy that promotes broader biomonitoring efforts to fully understand population variability in exposures, or interventions that promote more protective regulation or toxics use reduction.

## Conclusion

Much of the new biomonitoring work involves informing individuals of their chemical exposures, and the proliferation of individual report-back approaches discussed here represents a departure from traditional models of reporting aggregate study results in ways that are limited to academic settings, such as professional meetings and peer-reviewed publications. Of note is the increased effort among scientists to report chemical exposures whose clinical significance may not be fully known. There is a need for guidance on the ethical responsibilities associated with communicating individual and community-level data. Our research suggests that much of this guidance cannot solely come from the established arbiters of clinical and research practice, nor from government health officials, but must also include communities engaged in research collaboratives that have developed new standards of ethical report-back and participatory science practice.

## Abbreviations

CDC: Centers for Disease Control; NIOSH: National Institute of Occupational Safety and Health; EPA: Environmental Protection Agency; PBDEs: polybrominated diphenyl ethers; PFOA: perfluoroctanoate; PCBs: polychlorinated biphenyls; CBPR: community-based participatory research; EWG: Environmental Working Group; U.S: United States; IRB: Institutional Review Board.

## Competing interests

The authors declare that they have no competing interests.

## Authors' contributions

RMF, JGB, PB, and RAR originated the research to explore individual report-back in exposure studies. RMF conceived, designed, and implemented this study, led the writing, conducted some of the interviews, and analyzed interviews. JGB helped with the study design, analysis of interviews, and drafting of the manuscript. PB designed and implemented the study, analyzed interviews, and assisted with writing. RGA conducted most of the interviews, and participated in revisions of the manuscript. RAR assisted with analysis of the interviews and participated with the writing. CP assisted with interviews and provided critical input on the manuscript.
